# Imagery and perception: where is the phenomenological line?

**DOI:** 10.1192/j.eurpsy.2022.1821

**Published:** 2022-09-01

**Authors:** G. Simões, S. Jesus, R. Silva

**Affiliations:** Baixo Vouga Hospital Centre - EPE, Psychiatry And Mental Health Department, Aveiro, Portugal

**Keywords:** Psychopathology, Perception, Imagery, alterations of perception

## Abstract

**Introduction:**

The overlap between imagery and perception has long fascinated philosophers and scientists. Many scientists considered how the mind is capable of constructing an internal world without intervention of the external environment. Descriptions of their core characteristics often draw attention to differential features, but other currents reveal that many of these are shared rather than unique and differential.

**Objectives:**

The authors aim to analyse and discuss conceptualisation, similarities and differences of imagery and perception at the level of phenomenology, at the intersection with other psychopathological concepts, and thus reassemble them within a common framework.

**Methods:**

A brief literature review was developed based on relevant works containing subject matter most relevant to the topic.

**Results:**

Perception is conceived as a transformation of raw sensory stimuli into sensory information that is then decoded into meaningful at the cortical level. Imagery, in turn, corresponds to the internal mental representation of the world, actively drawn from memory. The differentiation between these concepts at a phenomenological level is analysed and discussed. Additionally, their individual role is evaluated in the pshycopathological expression of alterations of perception such as hallucinations, pseudohallucinations, pareidolic illusions, abnormal imagery, sensory deprivation and also of dreams, in an analytical perspective of integration and simultaneous conceptual differentiation.

**Conclusions:**

Understanding imagery, its nature and formal characteristics is required for better recognising the nature of perceptions and related psychopathological alterations, as well as the mechanisms uniting these concepts. Further research is needed as these entities represent features of useful clinical and diagnostic significance.

**Disclosure:**

No significant relationships.

